# On Metria (So-called Puerperal Fever)*This paper was prepared by Dr. Atthill, who was announced in the programme to open the discussion on the subject. He was, however, not present, and his paper was not submitted to the Section. It is published here to make the series of papers on the subject complete.

**Published:** 1883-09

**Authors:** Lombe Atthill


### On Metria (So-called Puerperal Fever).*

Your secretaries have requested me to initiate a discussion on the subject of Metria—a term which includes a number of affections which, though allied in some respects, are in others very dissimilar, but all of which are commonly classed together under the very incorrect title of "puerperal fever." I admit the force of their argument, that no more important or practical subject could be brought forward for discussion here; but I hesitated to accede to their wishes, because it is very recently that I read before the Obstetrical Section of the Dublin Academy of Medicine a paper on metria, which has already appeared in print, and from which my present communication cannot possibly materially differ. But, as your secretaries still consider it advisable that I should bring the subject under the notice of this Section of the British Medical Association on the occasion of its annual meeting, I consented to do so.

The pathology of metria is still far from being perfectly understood. Two facts alone are admitted by all who have

* This paper was prepared by Dr. Atthill, who was announced in the programme to open the discussion on the subject. He was, however, not present, and his paper was not submitted to the Section. It is published here to make the series of papers on the subject complete.

studied the subject carefully, namely: first, that puerperal women are liable, under certain circumstances, to be inoculated with septic matter conveyed to, and deposited in, the vagina, by the hands of the attendants, as well as by other agencies, when, either through carelessness or ignorance, proper precautions have not been adopted to prevent such an occurrence; and that the disease produced by such inoculation is not an unfrequent source of one of the forms of metria; secondly, that puerperal women may be self-inoculated by poisonous matter originating within their own bodies, from the decomposition of blood-clots formed within the uterus after parturition, or of portions of the membranes or placenta which have been retained *in utero*, the only difference of opinion on this point being, that Dr. Matthews Duncan and others term the disease thus produced "sapræmia"—that is, resulting from the absorption of putrid matter—thus distinguishing it from "septicæmia," or the disease produced by "organisms which, when conveyed to the blood, multiply indefinitely in it;" while those which are the product of putrefaction "do not survive, far less grow, therein." (Dr. Matthews Duncan on Puerperal Fever, *Lancet*, Nov. 6th, 1880.)

I hardly think that any one will dispute the correctness of the foregoing points; they have been established beyond all doubt; and it is certain that poison, introduced into the system by one of the two ways indicated, is the cause, in the vast majority of cases, of so-called puerperal fever, whether occurring in private or hospital practice. But there are many who believe that the whole subject is summed up in a belief of these very important propositions, and who think that to go outside these lines is only to cause difficulty and to create confusion. I admit this; but it seems to me that such an argument is almost an appeal *ad misericordiam*, and that it cannot be admitted for a moment. I believe that, in addition to the two preventable forms alluded to above, we have others; and I ask the members of this Section to consider whether we have not, in addition to these, two other forms of metria, which it may not be possible to guard against, namely:

1. A form of self-infection, occurring under special conditions, to which I shall allude by and by, which is not preventable by the adoption of any antiseptic treatment.

2. An epidemic, highly infectious form, which spreads by the same means as ordinary epidemics do.

Before commencing the discussion of these propositions, it is essential to bear in mind that I entirely concur in the opinion now generally held, that septicæmia, occurring in a puerperal woman, is not capable of being communicated to another puerperal patient by any means other than the direct transfer of the infectious matter to some portion of the mucous membrane lining the genital track. Septicæmia, however, when it attacks a puerperal woman, may be spread by various agencies, as well as by the hands of the attendant—for instance, by the nozzle of a syringe, by the use of infected sponges, by imperfectly washed napkins, bed-linen, etc.; but not through the medium of the air breathed by the patient. Of the truth of this I have not the slightest doubt.

You will observe that I have spoken of the two ordinary forms of puerperal septicæmia as being preventable. It is evident that, with thorough cleanliness, and the use of antiseptic precautions, septic poison should never be introduced into the patient's system by the attendants; further, I believe that it is possible to prevent self-infection in a healthy woman, by adopting precautions to insure a good and permanent contraction of the uterus, and by washing out the uterus whenever we have reason to suspect the existence of clots, etc., in it, with a disinfecting fluid. With the former object I make it a practice to put all patients in whom a relaxed condition of the uterus exists, on ergot, from the moment labor terminates, continuing its administration for at least a week. I believe a relaxed condition of the uterus to be a very common predisposing cause of self-infection in puerperal women; it favors the formation of clots, *in utero*, and also, the orifices of the uterine sinuses being left open, the absorption of septic matter is favored.

In proof that I do not exaggerate the importance of imperfect contraction of the uterus, as a main factor in the production of puerperal septicæmia, I may point out that I recently saw, in consultation, three patients suffering from this affection, in all of whom labor had been so rapid that the child was born before the arrival of the medical attendant; and, it is a well-known fact, that relaxation of the uterus is very liable to follow the too rapid emptying of that organ.

This train of reasoning has led me to believe that imperfect uterine contraction is one of the causes of the frequent occurrence of septicæmia in unmarried women. The mortality from septicæmia amongst them is very great, and there is no doubt but that the great mental distress these poor creatures suffer interferes with the recuperative process which should take place rapidly in the uterus after parturition. The muscular fibres of the organ do not contract as they should; the blood-supply, consequently, is not cut off, the mouths of the sinuses remain open, the denuded placental site, instead of becoming rapidly restored to its normal condition, becomes unhealthy, and the fœtid discharge, which, under these circumstances, takes the place of the normal lochia, enters the system, either directly through the open mouths of the placental sinuses, or is absorbed at the site of some fissure in the mucous membrane lining the genital track. This is one form of puerperal septicæmia which I fear is beyond the reach of preventive treatment. No antiseptic precautions can prevent its occurrence, no treatment that I know of will stay its progress. In patients suffering from certain forms of chronic disease a similar condition is observed, and similar results follow.

In my opinion, the infection arising from any of the forms of metria to which I have alluded, cannot be carried by the attendants from one patient to another, if precautions be adopted to prevent it. And only a year ago I was strongly inclined to believe that epidemics of so-called puerperal fever would not occur so long as such precautions were adopted. Those enforced by me among the pupils attending the Rotunda Hospital were the following:

1. Students attending the practice of the hospital should not undertake post mortem examinations, be engaged in dissections, or attend an hospital containing patients suffering from infectious diseases; and

2. Before proceeding to examine any patients, they washed their hands in a solution of carbolic acid. During the first six years and a half of my mastership, these sufficed to prevent the occurrence of anything like an epidemic of so-called puerperal fever. Deaths from septicæmia, especially among unmarried women, from time to time occurred, but the disease never spread. In August last, however, the hospital being at the time extremely healthy, a patient was admitted who complained of pain in the abdomen, and who vomited constantly, the fluid ejected being greenish. She stated that she had been in labor for more than twelve hours, and that, during the whole of that time, she had been vomiting; and it was subsequently elicited that she had been complaining for some days previously, and also that she had been seen, at the commencement of labor, by some practitioner, who advised her to go into hospital. The os, at the time of admission, was about one-third dilated; labor progressed very slowly, and she finally was delivered by the forceps. Vomiting ceased after delivery for a time, but soon recurred, everything swallowed being ejected, with large quantities of greenish fluid. The abdomen became tympanitic, the pain intense, matters went from bad to worse, and she died on the fourth day after delivery. Her appearance strongly resembled that of a patient suffering from typhus fever.

Another patient was admitted on the same day as the last patient, and she lay for a short time in the bed next to her. This patient's labor also was slow, but it terminated by the natural efforts. She was attacked with symptoms of acute peritonitis thirty-six hours after delivery, and almost immediately afterwards we noticed a very peculiar, almost black appearance of the face. The course of the disease was identical with that of the preceding case, but was even more rapid. The first symptoms showed themselves on the morning of the 29th, and she died on the 31st.

The disease now spread rapidly, and so virulent was the epidemic that out of twenty-nine women admitted during six days which intervened between the delivery of the first patient and the issue of the order to refuse admission to all applicants, eleven women were attacked and nine died.

The admission of patients being stopped, the wards were thoroughly disinfected, the walls lime-washed, the floors washed with a strong solution of chloride of lime, the cupboards, presses, etc., scoured, the nurses' clothes, as well as their bedding, being washed and aired, and placed in the hot-air chamber. Patients were re-admitted on September 12th; and from that date until the expiration of my mastership, on November 4th, during which time 118 women were admitted into the hospital, the health of the patients was excellent, and, I am informed, continues to be so still. No more successful effort to stamp out disease than this was ever recorded. This, and the fact that the epidemic was distinctly imported into the hospital, and that it did not originate in it, are facts as important as they are satisfactory: and though the occurrence of the outbreak was a cause of great distress to me, and though it was a great disappointment that, at the very close of my mastership, such a misfortune should have happened, still these two facts lessened the regret I naturally experienced.

Some years previously, a patient suffering from erysipelas of the head and face was, during the night, sent up to the labor-ward, her condition not having been detected till she was being undressed. The child's head was in the perinæum, and she could not be sent out. She was at once removed to a separate ward, and early next morning transferred to a fever hospital; but though her stay in the Lying-in Hospital was so short, several patients were attacked, not with erysipelas, but with so-called puerperal fever, and one died. The disease was limited to the one ward. I ask you, gentlemen, to consider what the disease attacking these women was. To me it seems to have been a disease originating by the introduction into the system of a puerperal woman of the infection of erysipelas, which infection was modified by the peculiar state of the system and of the blood which exists in puerperal women, and which, therefore, developed an apparently different disease; and I am strongly inclined to the belief that outbreaks of so-called puerperal fever, when it assumes an infectious and epidemic form, are due to the introduction of the poison of some ordinary zymotic disease into the system of a puerperal patient, the symptoms being, under such circumstances, totally different from those occurring in cases of septicæmia.—*Lombe Atthill, M. D., in British Medical Journal.*

